# Ice stream motion facilitated by a shallow-deforming and accreting bed

**DOI:** 10.1038/ncomms10723

**Published:** 2016-02-22

**Authors:** Matteo Spagnolo, Emrys Phillips, Jan A. Piotrowski, Brice R. Rea, Chris D. Clark, Chris R. Stokes, Simon J. Carr, Jeremy C. Ely, Adriano Ribolini, Wojciech Wysota, Izabela Szuman

**Affiliations:** 1 Department of Geography and Environment, School of Geosciences, University of Aberdeen, Elphinstone Road, Aberdeen AB243UF, UK; 2 British Geological Survey, Edinburgh EH93LA, UK; 3 Department of Geoscience, Aarhus University, DK-8000 Aarhus C, Denmark; 4 Department of Geography, University of Sheffield, Sheffield S102TN, UK; 5 Department of Geography, Durham University, Durham DH13LE, UK; 6 School of Geography, Queen Mary University of London, London E14NS, UK; 7 Dipartimento di Scienze della Terra, University of Pisa, 56126 Pisa, Italy; 8 Faculty of Earth Sciences, Nicolaus Copernicus University, 87-100 Toruń, Poland; 9 Department of Geomorphology, Adam Mickiewicz University, Poznan 61-680, Poland

## Abstract

Ice streams drain large portions of ice sheets and play a fundamental role in governing their response to atmospheric and oceanic forcing, with implications for sea-level change. The mechanisms that generate ice stream flow remain elusive. Basal sliding and/or bed deformation have been hypothesized, but ice stream beds are largely inaccessible. Here we present a comprehensive, multi-scale study of the internal structure of mega-scale glacial lineations (MSGLs) formed at the bed of a palaeo ice stream. Analyses were undertaken at macro- and microscales, using multiple techniques including X-ray tomography, thin sections and ground penetrating radar (GPR) acquisitions. Results reveal homogeneity in stratigraphy, kinematics, granulometry and petrography. The consistency of the physical and geological properties demonstrates a continuously accreting, shallow-deforming, bed and invariant basal conditions. This implies that ice stream basal motion on soft sediment beds during MSGL formation is accommodated by plastic deformation, facilitated by continuous sediment supply and an inefficient drainage system.

## Abstract (web summary)

Ice streams are fundamental to ice sheet dynamics, but the mechanisms controlling their flow remain elusive. Here, the authors perform macro- and microscale analyses of mega-scale glacial lineations, which indicate a continuously accreting, shallow-deforming bed during ice stream flow.

## Graphical abstract

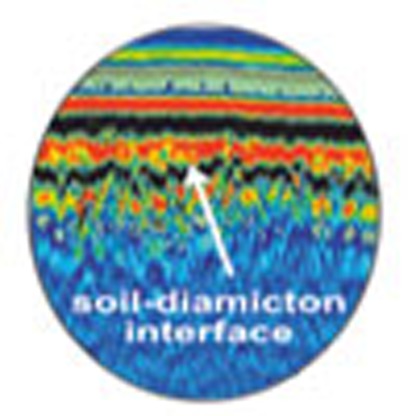

Ice streams play a fundamental role in the mass balance of ice sheets^1^. They have been referred to as the arteries of an ice sheet, because they can discharge >90% of their mass flux^2^,^3^. Model predictions of ice sheet response to atmospheric and oceanic forcing and associated sea-level fluctuations could be greatly improved by a more complete understanding of ice streams and their mechanisms of flow. Rare glimpses of ice stream beds, through geophysical and borehole observations^4^, have led to two possible explanations of the mechanisms governing ice stream flow: (i) basal sliding facilitated by water pressures at overburden^5^,^6^, with the ice stream effectively decoupled from its bed^7^, and (ii) basal motion accommodated via deformation of either thick (several metres)^8^,^9^ or thin (centimetres to decimetres)^10^,^11^ layers of the underlying ‘soft' sediments. Resolution of this debate has fundamental implications for subglacial sediment erosion, transport and deposition. A better understanding of processes at the ice stream bed could also lead to the development of more sophisticated and robust models of ice stream flow dynamics and, ultimately, ice sheet mass balance and sea-level change. For example, recent modelling has highlighted that the relationship between basal friction and sliding is a key ‘unknown' when attempting to model Antarctica's future contribution to sea-level rise^12^.

When ice stream beds are associated with the presence of soft sediments, they are typically organized into corrugations known as mega-scale glacial lineations (MSGLs)^13^. These extremely elongated landforms have been observed evolving under an Antarctic ice stream^14^ and are common along palaeo-ice stream troughs proximal to the present day Antarctic ice streams^15^ and in numerous palaeo ice-sheet settings as well, both onshore and offshore^16^,^17^. As MSGLs are produced at the ice stream bed, an analysis of their sedimentary properties can contribute to the debate on their genesis^18^,^19^,^20^ and advance understanding of ice stream motion by potentially distinguishing between basal sliding and bed deformation as a mechanism of fast flow.

During the last glaciation, the SE sector of the Scandinavian Ice Sheet covered much of the Baltic region and was drained by a series of ice streams^16^,^21^. This study focuses on the Odra palaeo-ice stream (OPIS), located in Poland near the city of Poznań, close to the ∼21 ka Leszno phase ice margin, representing the local last glacial maximum^22^,^23^. The bed of the OPIS, exposed across a region of over 1,000 km^2^ in the Wielkopolska Lowland, is underlain by a thick (∼30 m) sequence of Quaternary sediments and represents one of the few regions in onshore Europe to show a well-preserved assemblage of MSGLs.

The OPIS MSGLs are characterized by the same long axis orientation (∼130°N), a regular spacing (crest-to-crest distance) of 500–700 m, and a generally low relief of 2–4 m (Fig. 1), which is consistent with previous measurements from a variety of ice stream beds^24^. Some of the MSGLs can be traced continuously for over 16 km and they are thought to have been originally much longer, with deglacial meltwater channels and the extensive urbanization of Poznań interrupting their continuity^23^.

Here we present a suite of detailed sedimentological analyses from ten sites located across the best-preserved part of the OPIS MSGL field, including ridge crests and flanks. Results reveal, at all sites and depths, that the sediment has near-identical granulometry, strong and consistent macro- and microfabric, and similar petrography, whereas the stratigraphy is represented by a single massive unit of silty–sandy diamicton. The homogenization of the OPIS bed and the fine-grained nature of the sediment indicate ice stream basal conditions dominated by continuous sediment accretion and shallow, but pervasive, deformation during the formation of MSGLs.

## Results

### Stratigraphy and sedimentology

**Figure 1:**
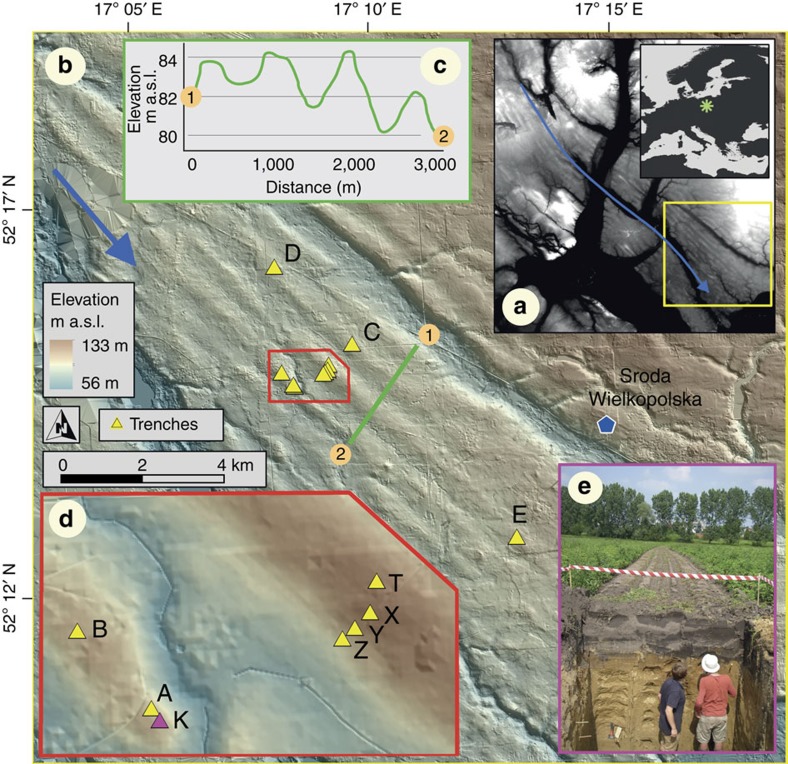
Map and location of sample sites. The OPIS bed, Poland (**a**). It comprises a series of MSGLs indicative of a NW–SE ice flow (blue arrow) with considerable elongation (**b**) and is characterized by very low relief (**c**). Ten trenches (labelled with capital letters) (**b**,**d**) along the crests and flanks of the MSGLs were opened and analysed in detail (**e**, showing site K).

With the exception of two relatively small structures (one ice wedge cast at site T and some rootlets at site D), all ten sites present an identical stratigraphy, with only slight variations in the modern soil depth, typically 0.3–0.5 m from the ground surface (Fig. 1e). The sediment body comprising the bulk of the MSGLs' relief is characterized by a homogeneous single unit of massive, matrix-supported, silty–sandy diamicton, lacking any evidence of outcrop-scale glaciotectonism (for example, thrusting and folding; Supplementary Fig. 1). The diamicton appears yellow, apart from a few rare patches where calcification has occurred, usually affecting areas <200 cm^2^. Gravel-sized clasts (2–64 mm) are rare and cobbles (>64 mm) are extremely rare. At no site was any other sedimentary unit exposed.

**Figure 2:**
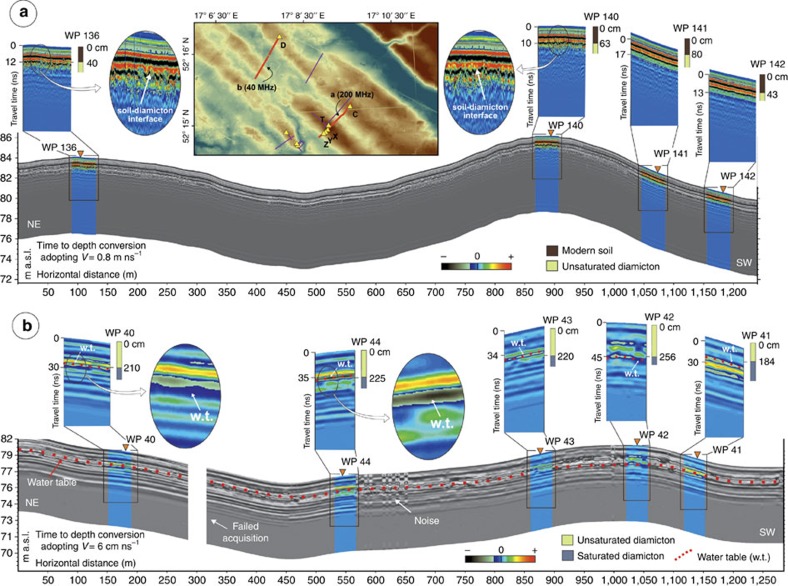
Two GPR profiles from the 200 and 40 MHz surveys. Details are enlarged and coloured in correspondence to the auger samplings (WP) and, in two cases, further enlargements show the strongest reflectors identified within each profile. In **a**, the only significant reflector besides the surface waves is found at a variable depth of 0.3–0.5 m, and augering and trench observations confirm this to be the boundary between the organic soil and the diamicton below. In **b**, the only significant reflector is verified at a depth of 1.8–2.6 m and augering confirms this to correspond to the water table/capillarity fringe system within the diamicton. All other reflectors, parallel to the surface or the water table, are interpreted as multiples.

The ground penetrating radar (GPR) reached the water table (typically 2–3 m) and, with the exception of a few infilled palaeo-channels and small oblique and discordant reflections, interpreted as ice wedge casts, the >10 km of GPR lines revealed a uniform radar stratigraphy. This indicates that the trench data are representative of the MSGL field. All 200 MHz acquisitions, an example of which can be seen in Fig. 2a, show a series of surface waves followed by only one clear subsurface reflection, the depth of which corresponds to that of the soil base. This reflection, almost perfectly parallel to the surface, indicates the stratigraphic change from the organic, aerated soil above to the diamicton below. The slight irregularity of this interface, the depth of which varies from 0.3 to 0.5 m from the ground level (verified by augering and observations in the trenches), is most likely to be due to agricultural activity. No other reflections are evident below this interface, suggesting either a complete absence of structures or a lack of penetration or resolution. Given the sandy nature of the sediment, it is unlikely that penetration was limited to only the first 0.3–0.5 m of the profile. A complete lack of structures at greater depths is confirmed by all 40 MHz acquisitions (for example, see Fig. 2b). Besides the usual surface waves and their multiples, and the soil–diamicton interface reflector and its multiple, all 40 MHz profiles reveal no other reflector to a depth of ∼2–3 m where a series of strong reflectors are generated corresponding with the water table and capillary fringe system (verified by augering and observations in the trenches). This follows the same geometry as the topography, although it becomes slightly shallower in the deepest part of the profiles (MSGL troughs).

### Macroscale fabric

**Figure 3:**
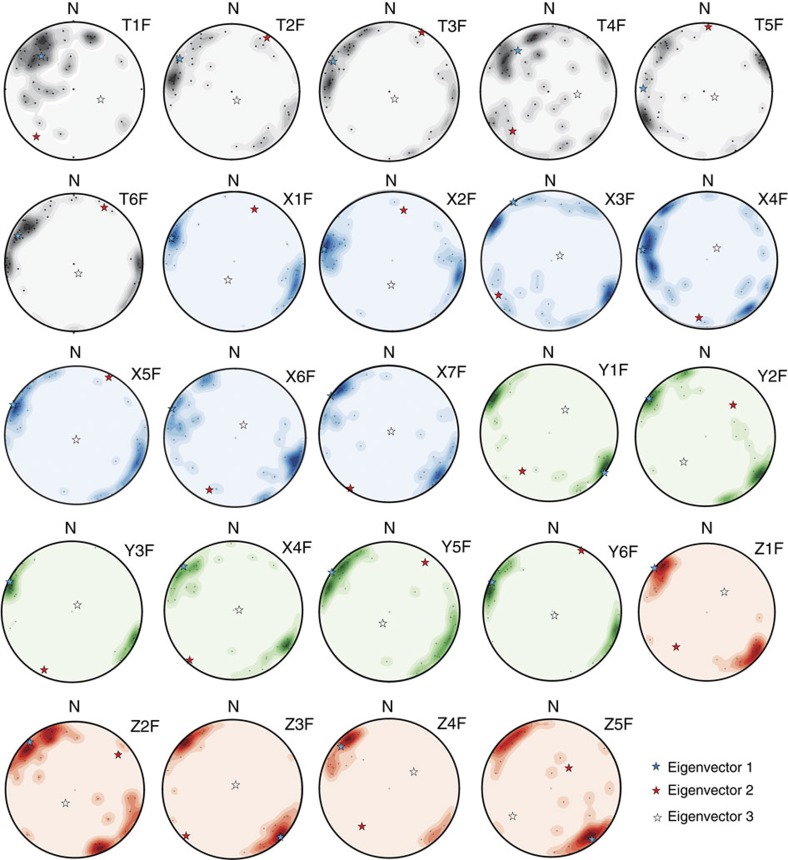
Stereoplots of clast macrofabric collected at key sites across the flank of the same MSGL. At all depths and sites the same strong fabric is evident, characterized by a NW–SE orientation and a dip angle of less than 10°. Eigenvectors are visible as stars in the main plots. Samples are progressively numbered according to their relative depth position, from the top of the diamicton (for example, T1F), close to the soil–diamicton boundary, to the deepest portion of the diamicton reached at each trench (for example, T6F). Eighty per cent of all T, X, Y and Z *S*_1_ eigenvectors are within 121(301)±18°N.

**Figure 4:**
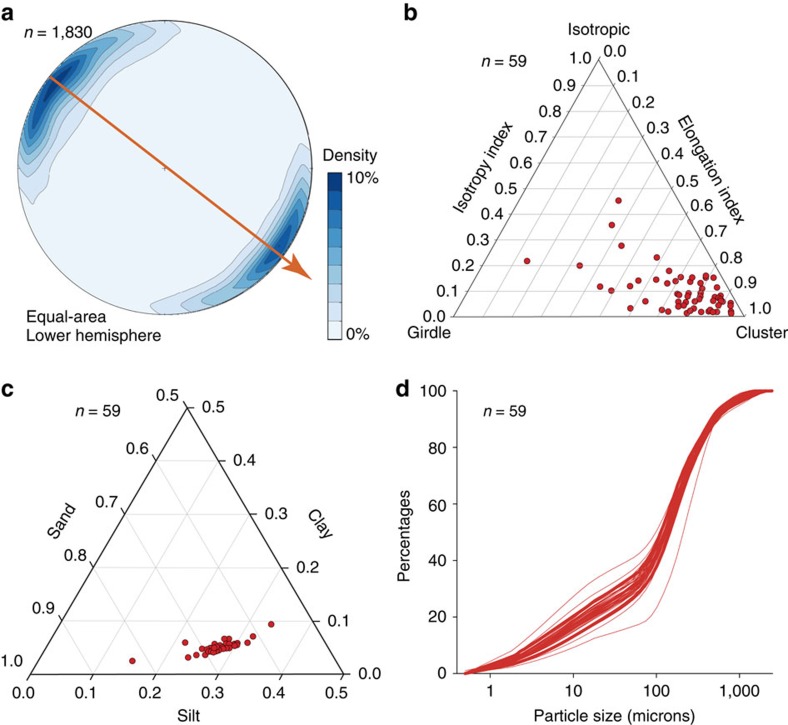
Key sedimentological properties of the investigated sites demonstrating consistent fabric and granulometry. Panel (**a**) demonstrates that most clasts indicate a dominant NW–SE orientation, similar to that of the MSGL long axes (orange arrow), whereas (**b**) shows that most samples are characterized by a clustered fabric. Panel (**c**) demonstrates that the sediment is consistently a sandy silt, whereas (**d**) shows the consistency of the frequency distribution at various particle size intervals (below 2 mm).

Clast *a*-axis macrofabric was measured at multiple depths in ten sites across the OPIS bed, including some that are kilometres apart along the same MSGL crest (for example, site T and E) and others that are distributed across the same MSGL flank, from near the trough to the crest (site T, X, Y and Z). The azimuth and dip of a minimum of 30 clasts, with an elongation ratio ≥2:1 (*a*:*b* axes) and typically *a*-axis in the range of 5–20 mm, were measured from an area of ∼30 × 10 cm^2^ at each depth interval. Clast macrofabric along the crest of, or across, the same MSGL was found to be similar, with no evidence of any systematic variation horizontally and vertically (such as a herringbone pattern) (Supplementary Table 1 and Fig. 3). Macrofabrics are generally consistent across all sites and depths, showing shallow dips and a dominant NW–SE direction, concordant with MSGL long axis orientation (Supplementary Table 1 and Fig. 4a). Eighty per cent of all *S*_1_ eigenvectors are within 121.5(301.5)±11.5°N. The normalized eigenvalues of the macrofabric data are very high, with a mean value of 0.75. The vast majority of fabric shapes, derived from the ratios between the three main eigenvalues plotted on an equilateral ternary diagram^25^, is concentrated on the cluster apex (Fig. 4b). This indicates a very low isotropic index (that is, observations confined to a single plane or axis) and a very high elongation index (that is, a strong preferred orientation and most observations parallel to each other).

### Thin section analysis

**Figure 5:**
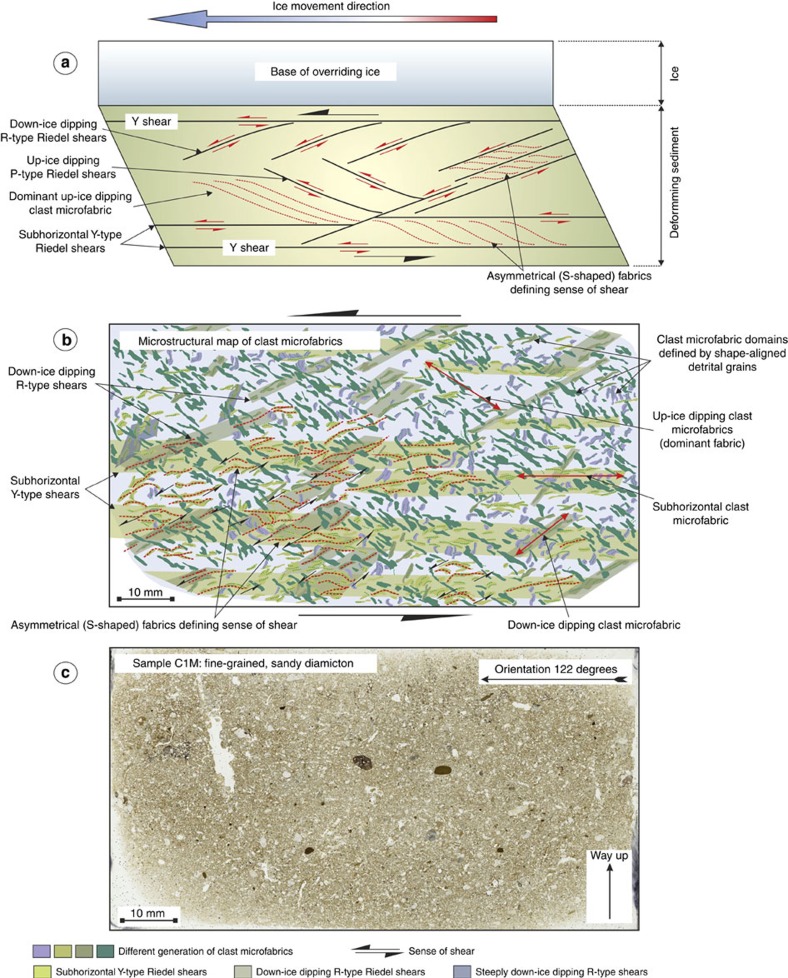
Microstructural map of thin section C1M. (**a**) Diagram showing the relationships between the different sets of Riedel shears developed within the diamicton in response to deformation imposed by the overriding ice stream; (**b**) example of a detailed microstructural map of a thin section of diamicton from site C. The coloured polygons represent the different generations of clast microfabrics, which define the Riedel shears, subhorizontal shear fabric and up-ice dipping foliation, and (**c**) high-resolution scan of sample C1M highlighting the massive, fine-grained sandy nature of the diamicton.

Microscale analysis of orientated thin sections of the diamicton within the MSGLs reveals a complex, but systematic, array of deformation fabrics, which can be interpreted as having formed by the passive rotation of sand-grade particles, into the planes of the foliations, defining a number of clast two-dimensional (2D) microfabrics^26^,^27^. Initial analysis of all the thin sections revealed that the composition, texture and structure of diamicton are uniform across the study area. Consequently, subsequent analysis of the microfabrics focused on site C, enabling any changes in the relative intensity and/or style of deformation, upward through the sediment profile, to be examined in detail. In thin sections, the diamicton at this site appears composed of fine- to medium-grained, matrix-supported, silty sand containing scattered, angular to well-rounded granule, to small pebble-sized rock fragments (limestone, granite, sandstone and schistose metamorphic rocks). Sand grains are mainly composed of monocrystalline quartz and subordinate amounts of feldspar and exhibit preferred shape alignments. The geometry of these microstructures in each thin section were analysed using a standard methodology^26^. An example of the resultant ‘microstructural map' is shown in Fig. 5. This analysis reveals that deformation was dominated by foliation development with the lack of folding and/or faulting. The clast 2D microfabrics define a conjugate set of Riedel shears, as well as a subhorizontal shear foliation (Fig. 5a,b). These probably developed in response to shearing imposed by the overriding ice, with the subhorizontal foliation having formed parallel to the base of the ice (see Fig. 5a). The geometry, orientation and kinematic indicators (for example, asymmetry of S-shaped 2D microfabrics) recorded by the fabrics (see Fig. 5a,b) are consistent throughout the sediment profile and record an SE-directed sense of shear, coincident with the long axes of the MSGLs and the regional ice flow pattern.

A prominent, subvertical foliation present within the lower part of the diamicton sequence locally overprints the earlier shear fabrics and is interpreted as recording the subsequent dewatering of the sediments within the core of the MSGL. Dewatering and consolidation would have been driven by the ice overburden pressure. This may have occurred penecontemporaneous with landform development, or shortly after the cessation of fast ice flow when the diamicton was unconsolidated and still able to respond to the dewatering.

### 3D-computed X-ray microtomography analysis

**Figure 6:**
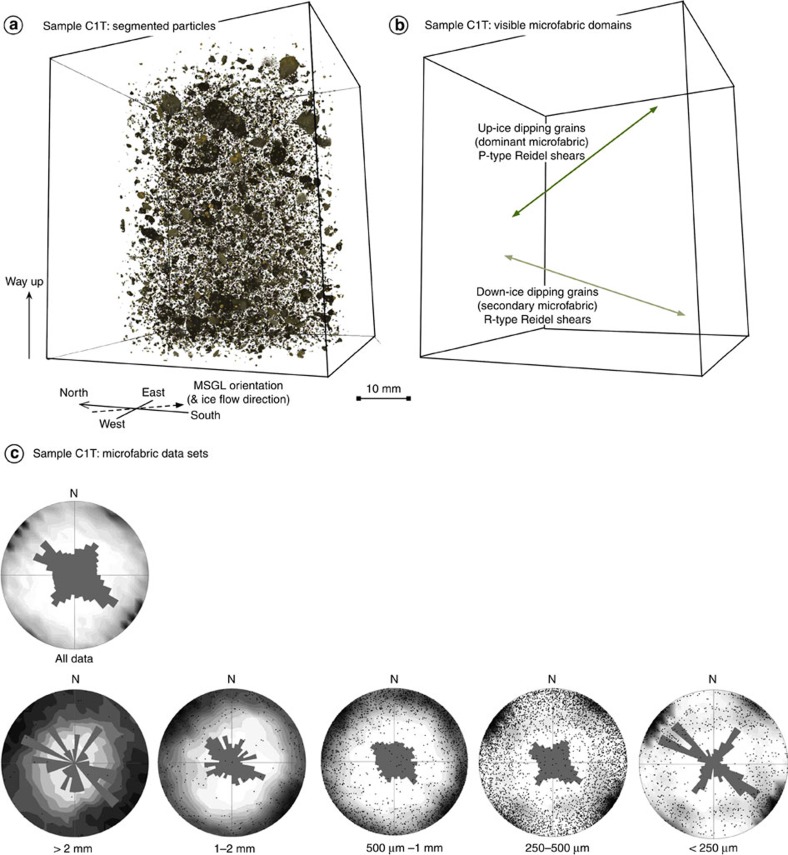
Visualization and fabric analysis of μCT sample C1T. (**a**) Visualization of the segmented bulk phase interpreted as skeleton particles. Within what is a complex arrangement of particles (*n*≥120,000), it is possible to identify chains of particles reflecting two geometries, one dipping up-glacier by ∼24° and the other dipping down glacier at ∼10° (it is noteworthy that the angles look steeper in the image due to the transposing of a 3D volume onto a 2D surface). (**b**) Identification of the two main particle chain geometries, which are interpreted as representing the P- and R-type Riedel sets identified in thin section. (**c**) Rose diagrams and contoured stereoplots of particle fabrics (aspect ratio >1.5:1) from sample C1T. The large data set of fabric analysis permits the partitioning of particle fabric data by particle size and identifies that the multiple modes recorded in the full fabric data are a consequence of systematic orientation of particle size fractions either parallel or transverse to ice-flow direction. Original rose diagram eigenvectors and eigenvalues can be found in Supplementary Table 2.

The three-dimensional (3D) visualization of the particle bulk phase of all X-ray microtomography (μCT) scanned samples highlights that within a complex overall fabric signature, two distinctive geometries are represented by chains of particles (Fig. 6a). The dominant geometry is of discrete planes of particles with *a*-axes dipping apparently up-glacier at ∼24° relative to the horizontal, whereas the second geometry has a more variable (mean of ∼10° to the horizontal) down-ice dip (Fig. 6b). These compare well with the two main Riedel shear geometries identified in vertical thin sections (noted above). Quantification of particle fabrics from all scanned samples (a typical example of the data is shown in Fig. 6c) illustrates a distinctive bimodal pattern, with the main modes parallel to MSGL orientation (and inferred ice flow direction). Distinctive secondary modes are oriented transverse to inferred ice-flow direction and are most strongly developed in the finer particle fractions (*b*-axis <500 μm). Low resulting *V*_1_ dip angles are a statistical artifact of eigenvector analysis of samples with multiple fabric modes. Derived eigenvalues remain broadly consistent between samples, representing a strong girdle fabric shape in all samples. The geometry and kinematics recorded by the μCT data sets are spatially consistent: vertically within the sample, vertically within each trench and between sites, and record a sense of shear that is parallel to the orientation of the MSGLs and inferred ice-flow direction.

### Petrography and granulometry

**Figure 7:**
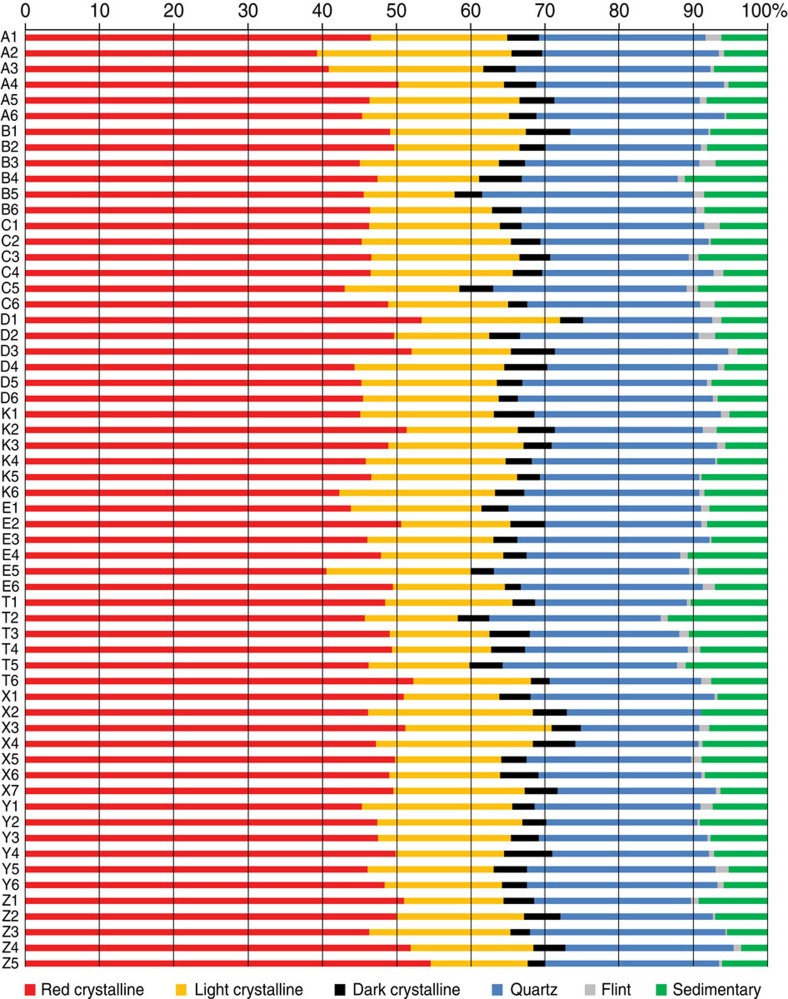
Petrographic composition of all samples A1 to Z5. The diagram, which shows the content of weathering-resistant components at every site (per letter code, y-axis) and depth interval (per number code, increasing depth, y-axis), demonstrates consistency across and within sites.

The clast (2–4 mm) petrography was determined on a minimum of 300 grains per depth interval, with a distinction made between weathering-resistant components including sedimentary, flint, quartz and (red, light and dark-coloured) crystalline lithologies, and components susceptible to postdepositional weathering. Overall, the composition of lithologies is consistent across all samples (Fig. 7). Within the weathering-resistant components, red crystalline lithologies vary from 39 to 54%, light crystalline from 13 to 21% and dark crystalline from 2 to 5%. The quartz component comprises between 15 and 28%. Flint is always <3%, whereas sedimentary components account for between 3 and 11%. A detrended correspondence analysis shows minimal variability in terms of s.d. units (axis 1=0.28 and axis 2=0.24) and a principal component analysis indicates that Euclidean distance, in multidimensional space, between the samples is very small and no depth or site clustering pattern is revealed. The total composition includes Palaeozoic limestone sourced from the Baltic Basin and crystalline rocks from the Scandinavian Shield, thus indicating a far-travelled origin for much of the glacial sediment. Similar compositions have been found in Germany and Denmark^28^,^29^,^30^, and indicate deposition by ice of north-easterly provenance.

The <2 mm fraction of all diamicton samples is largely composed of sand (62–71%) with a minor component of silt (29–38%) and low clay contents (4–9%) (Fig. 4c). Grain size distributions are also consistent, even between sites as far apart as 6 km; within any particle size interval, the relative frequency spread is less than 5% (Fig. 4d). No granulometric trends were found either vertically or horizontally across all sites.

## Discussion

In summary, ten sites were investigated and sampled at a high vertical resolution, within the same MSGL and across multiple MSGLs, and there are no obvious differences in clast macro- or microfabric (orientation and strength), petrography and granulometry. Sediment homogeneity might be responsible for the lack of visible evidence of outcrop-scale thrusting or folding, as these are difficult to identify when they do not involve deformation of distinctively different materials. However, given the density of sampling, a variation in fabric should have been evident had faulting or thrusting been present. Moreover, based on observations from extant^9^,^31^ and palaeo^32^ ice stream beds, diamictons are typically porous and weak, with the water content close to the liquid limit and therefore precluding folding or thrusting.

The preservation of the OPIS MSGLs, coupled with the homogenous and massive architecture of the diamicton, and the rare presence of postformational periglacial, glaciofluvial and fluvial disturbances demonstrates that these landforms and their internal structure reflect basal processes occurring beneath the active ice rather than in ice-marginal or proglacial settings. The vertical and horizontal consistency of the clast macro- and microfabrics indicates that the diamicton has experienced pervasive shearing. Theoretical^10^, experimental^33^,^34^ and empirical data^7^,^11^,^35^ indicate that the depth of deformation in (Coulomb plastic) diamicton is likely to be less than a few decimetres. Pervasive deformation to greater depths could theoretically be achieved under three conditions: ploughing by clasts held in the basal ice^11^, bridging across grain networks^36^,^37^ or shearing zone migration due to water pressure fluctuations^10^. All three conditions require the presence of a coarse-grained diamicton and/or large clasts, neither of which are found in the OPIS bed. Furthermore, under conditions of clast ploughing or grain bridging, a thick deforming layer should theoretically display a decreasing-with-depth strain profile, which could be expected to be detected by changes in granulometry, petrography or fabric strength; however, this was not found. As such, our interpretation is that ice stream flow over the sediment was sustained by pervasive deformation in a thin shearing zone, with the >1.4-m-thick homogenized diamicton being the product of continuous subglacial accretion^38^. Under these conditions, the homogeneity of the sediment body and the lack of outcrop scale glacitectonism suggest a constant supply of sediment and largely invariant boundary conditions such as basal water pressure, basal temperature and sediment strain rate.

Two possible scenarios might be envisaged to link the sedimentary processes to the formation of the MSGLs. One scenario is that pervasive deformation of the bed was concomitant with the formation of the MSGLs, with the implication that the origin of these landforms is constructional during bed accretion. The other scenario is that the strain signature was previously imposed on the sediment and a later phase of ice stream flow generated the MSGLs by erosion. The sedimentological characteristics (weak and inconsistent fabric, facies variability and the presence of sediment rafts) found on at least one palaeo ice stream bed^39^, resting on the hard bedrock of the Canadian Shield, partially support the erosional hypothesis. However, none of these characteristics have been verified in the OPIS bed, which rests on a thick sequence of ‘soft' Quaternary deposits. Some theories of MSGL formation have advocated erosion of the bed either by ice keels (groove-ploughing theory^18^) or water flow (megaflood^19^ or rilling instability^20^ theories). Sites T, X, Y and Z represent a transect from the crest to near the bottom of the trough and show the same sediment granulometry, demonstrating no depletion of fines related to flowing water, even towards the base of the troughs. Combined with an absence of any meltwater-related deposits, these observations question the idea of MSGL formation by flowing subglacial water or rilling erosion. The groove-ploughing theory^18^ suggests that basal ice keels are formed either by ice streaming over rough bedrock upstream of the MSGLs or by an area of flow convergence. However, the thick sequence of Quaternary sediments in the studied region must have precluded the formation of bedrock-related ice keels and detailed reconstructions provide no evidence of ice flow convergence^21^,^23^. The theory also suggests that during the formation of the MSGLs, sediment is redistributed from the landform trough to the flanks. As diamicton is squeezed laterally into intervening ridges, a herringbone signature is generated in the fabric^18^. Although it is possible that the greater strain in the downflow direction might partially mask it, no evidence of any systematic variation in fabric was found across the OPIS MSGLs, vertically or horizontally, both at the macro and at the microscale. The groove-ploughing theory also predicts that MSGLs width increases and their height decreases downstream, as ice keels melt due to frictional heating^18^. However, the (modest) morphometric variability of the OPIS MSGLs shows no evidence of downstream changes. Taken together, these observations provide little support for the formation of the OPIS MSGLs via an erosional mechanism, thus suggesting that pervasive deformation of the bed was concomitant with the evolution of the MSGLs. Indeed, micro and macrofabric, at all sites and depths, have the same orientation as the MSGL main axes. Had the strain signature been previously imposed on the sediment, this correspondence would require the earlier ice flow to have had the exact same orientation of the subsequent (topographically unconstrained) ice stream that eroded the MSGLs. Instead, the observations are more easily explained by sediment deposition being coeval with landform shaping, with MSGLs representing constructional features.

Although the actual process of moulding the bed into the ice-flow parallel ridges and troughs (MSGLs) remains elusive, the data presented here indicate that they were formed through continuous sediment accumulation. Thus, to generate an uneven topography, a higher rate of accretion must have selectively occurred towards the crests of the MSGLs. Significantly, this study demonstrates that the OPIS MSGLs record ice stream flow via thin-skinned deformation, under largely invariant sedimentary and hydrological basal conditions. Specifically, indicative of an inefficient, distributed drainage system are the continuity of the MSGLs with a lack of evidence for a major meltwater drainage network; the homogeneity of the diamicton, that is, no depletion of fines; and the absence of deformational structures related to water pressure fluctuations and eluviation^40^.

The MSGLs analysed here are morphologically similar to those of many other settings worldwide^24^. Their sedimentology is also compatible with most other studies of soft ice stream beds^41^ and, in particular, with the geophysical and borehole observations of an unconsolidated, porous diamicton corresponding to the acoustically transparent seismic horizon that characterizes most Antarctic ice stream beds^32^,^42^. Given the widespread presence of MSGLs associated with soft-bedded ice streams, this work has fundamental implications for the interpretation and modelling of ice stream dynamics; in particular, ice stream basal motion on soft sediment beds is accommodated by plastic deformation of a thin layer of sediment and facilitated by continuous sediment supply and an inefficient drainage system.

## Methods

### Field work and macroscale analyses

Detailed investigations were focussed on ten sites located across the best-preserved part of the OPIS MSGL field, including ridge crests (sites A, B, C, D, E, K and T; Fig. 1) and flanks (T, X, Y and Z; Fig. 1). A trench 6- to 10-m-long, 2- to 3-m-wide and 3- to 5-m deep was opened at each site (Fig. 1). Field work was carried out in three campaigns during the summers of 2011, 2012 and 2013, whereas laboratory analyses were conducted in 2013 and 2014. Macro-sedimentological analyses were carried out on a free face, usually parallel to the MSGL long axis within each trench. The free face was initially cleaned, with a stratigraphic log and an annotated sketch of the section made. The face was then subdivided into 10-cm-thick sample sections at vertical intervals of 20 cm working from the base of the modern soil to the bottom of the trench, 1.2–1.4 m below, giving 5–6 intervals per site and 59 in total. Clast *a* axis macrofabric measurements were carried out on clasts with an elongation ratio of at least 2:1 (*a*:*b*). All visible elongated clasts were measured, typically with an *a* axis length range of 5–20 mm. Clasts were measured at each depth interval across an exposed surface of about 30 × 10 cm. The fabric measurements were undertaken by multiple operators at each site, with usually one operator per sampled interval. Samples were also collected for quantitative petrographic and granulometric characterization, which was carried out at the sedimentological laboratory of the Department of Geoscience, Aarhus University, Denmark. In addition, samples were collected for micromorphology and X-ray tomography analyses. Morphometric analysis of the MSGLs was carried out with ArcGIS on a 5-m resolution digital terrain model, using standard techniques^24^.

### Thin-section analysis

Samples for the thin sections were collected at all sampling sites and depths with standard kubiena tins. Thin sections, prepared using standard methods developed at the Centre for Micromorphology, Royal Holloway, University of London, were taken within approximately ±5° of the MSGL long axes. The cutting plane for thin sectioning was oriented parallel to the MSGL long axis. The thin sections were examined using a standard Zeiss petrological microscope. Detailed microstructural maps and quantitative data for the clast microfabrics developed within the diamicton were obtained by first scanning the thin sections at high resolution and then importing these into a computer graphics package.

### 3D X-ray μCT analysis

This technique permits the imaging of the properties of sediments at high resolution, recording variations in material density and atomic weight, which are partitioned by the user into bulk phases, each representing a different component of the sample^43^. In this study the focus has been on deriving 3D microscale particle fabric data, an example of which can be seen in Fig. 6. Samples for 3D X-ray μCT scanning were collected at all sampling sites and depths, recovering undisturbed samples within 60-mm-long, 40-mm-diameter plastic piping. Sealed samples were scanned on a Nikon X-Tek XT-H 225 Micro-CT system at the Centre for Micromorphology, Queen Mary University of London, with a voxel size of volumetric reconstructions of 62.5 μm. Three-dimensional microfabric analysis was undertaken on all data sets. The heterogeneity of glacigenic sediments analysed in this study offers particular challenges for the identification and segmentation of particles representing mixed mineral assemblages using μCT. Consequently, a machine-learning tool^44^,^45^ has been applied to data sets (*n*=53), enabling systematic, objective and robust identification of all particles with *b* axis >250 μm (Fig. 6a). Object-based analysis^46^ permits extraction of azimuth and dip of the *a* axis of all particles with an *a*:*b* axial ratio >1.5:1 (Fig. 6c). The resulting data sets overcome the sample size weaknesses associated with macroscale clast fabric analysis^47^, by generating large data populations (typically two to three orders of magnitude greater than for clast macrofabric) that can be partitioned and interrogated in detail (Fig. 6c).

### GPR analysis

GPR acquisitions were carried out along and across the MSGLs long axes, with profiles usually located near the trenches used for the sedimentological analyses, using an IDS Radar System (www.ids-spa.it). Acquisitions were made with a monostatic transmitting and receiving 40 MHz (nominal peak frequency) unshielded antennae and 200 MHz shielded antennae. A total of 12 GPR profiles were acquired, some as long as 1,200 m, covering a total surveyed length of 10,300 m; most profiles were repeated with both antennas. Data for the 40-MHz survey were captured in step collection mode with a step length of 1 m, whereas a continuous mode acquisition was adopted for the 200 MHz survey, with a step length of 0.25 m. Configuration for both data acquisitions provided 1,024 samples per scan in a time window of 200 ns. A standard processing sequence was applied to the raw data to adjust GPR traces to a common time-zero position, filter out noise and gain attenuated GPR signals. Specifically, a horizontal running average filter was applied to remove the saturation effect caused by Tx–Rx direct coupling. A subtraction of the mean trace to the data set (background removal) was applied to filter out continuous flat reflections caused by multiple reflections between the antenna, the operators and the ground surface. This filter was applied to the data from the 40 MHz survey and limited to the first 10 ns, to avoid disrupting reflections from continuous flat layers below the surface. Following a spectral analysis of measured signals, a band-pass filter (21-38-110-156) was applied, to remove undesired frequencies coming from instrumental and environmental noise. To enhance the visibility of deeper reflections due to signal attenuation, a gain function, increasing linearly with depth, was applied. The availability of water table depths along the GPR profile (measured by augering) allowed calibration and to convert the arrival times of reflected radar waves to depth below surface. Calibrations based on each auger sample, with an instrumental accuracy of ±15 cm, were consistent with each other and indicated an EM wave velocity of ∼6 cm ns^−1^, in line with the velocity usually defined for this type of sediment in unsaturated conditions. This value was used for the time-to-depth conversion for all 40 MHz radargrams. The existence, in the 200-MHz acquisitions, of some diffraction hyperbola allowed adoption of the synthetic hyperbola method. An estimated EM wave velocity of about 8 cm ns^−1^ was consistently found and applied to all 200 MHz profiles.

## Additional information

**How to cite this article**: Spagnolo, M. *et al.* Ice stream motion facilitated by a shallow-deforming and accreting bed. *Nat. Commun.* 7:10723 doi: 10.1038/ncomms10723 (2016).

## Supplementary Material

Supplementary Information Supplementary Figure 1 and Supplementary Tables 1-2.
