# Supplementary material for: Ice stream motion facilitated by a shallow-deforming and accreting bed
Source: Nat Commun. 2016 Feb 22;7:10723. doi: 10.1038/ncomms10723 (PMC4764869; doi:10.1038/ncomms10723)
Supplement: Supplementary Information — Supplementary Figure 1 and Supplementary Tables 1-2. [file ncomms10723-s1.pdf]

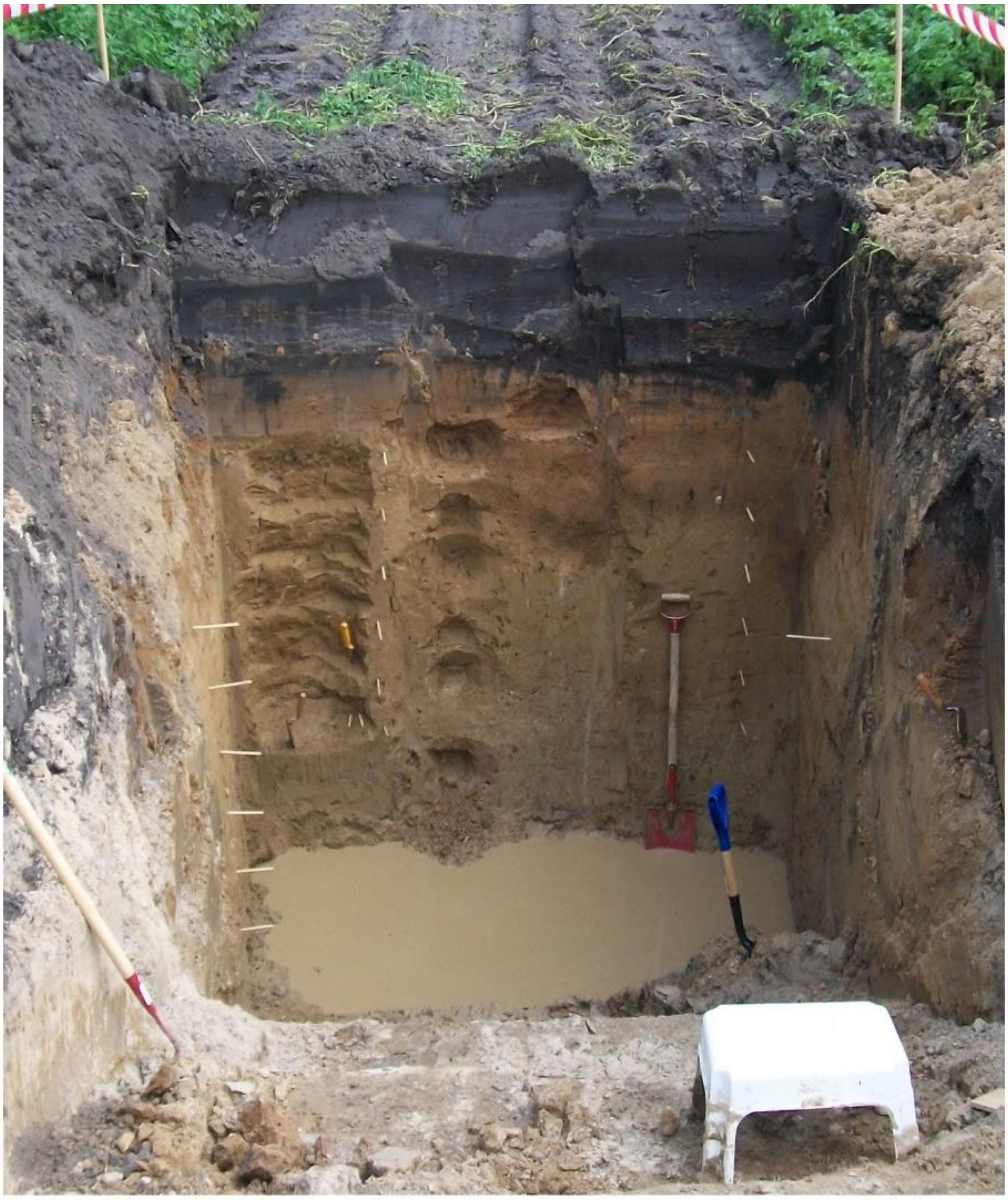

Supplementary Figure 1. **Site K.** Photograph showing the characteristic silty-sandy, massive nature of the OPIS diamicton, below a 2-3 dm dark soil horizon.

site	n	eigenvectors			normalised eigenvalues			Fabric (Benn, 1994)	
		1	2	3	S1	S2	S3	isotropy S3:S1	elongation 1-(S2/S1)
A1	30	316.0 / 4.3	46.2 / 3.9	178.1 / 84.2	0.841	0.1	0.06	0.071	0.881
A2	30	305.2 / 4.6	37.4 / 24.4	205.2 / 65.1	0.777	0.179	0.044	0.057	0.770
A3	30	339.4 / 7.3	248.4 / 8.2	110.5 / 79.0	0.774	0.211	0.016	0.021	0.727
A4	30	314.8 / 9.0	61.7 / 61.3	220.2 / 27.0	0.822	0.115	0.063	0.077	0.860
A5	30	319.4 / 15.8	85.6 / 64.4	223.6 / 19.6	0.689	0.205	0.106	0.154	0.702
A6	30	153.4 / 3.5	244.4 / 16.6	51.9 / 73.0	0.732	0.202	0.066	0.090	0.724
K1	30	329.9/5.3	235.7 / 37.6	66.7 / 51.9	0.851	0.095	0.054	0.063	0.888
K2	30	323.1 / 5.0	53.3 / 2.0	165.5 / 84.6	0.817	0.156	0.027	0.033	0.809
K3	30	330.9 / 9.7	62.7 / 9.9	197.3 / 76.0	0.784	0.181	0.035	0.045	0.769
K4	30	320.2 / 2.7	50.2 / 0.9	158.3 / 87.1	0.819	0.168	0.012	0.015	0.795
K5	30	323.7 / 7.7	233.0 / 5.5	107.8 / 80.6	0.817	0.163	0.02	0.024	0.800
K6	30	323.0 / 6.4	232.0 / 9.4	86.8 / 78.6	0.85	0.134	0.016	0.019	0.842
B1	30	323.7 / 1.2	53.9 / 7.0	224.0 / 82.8	0.833	0.115	0.052	0.062	0.862
B2	30	317.6 / 6.4	226.1 / 13.0	73.3 / 75.4	0.752	0.179	0.069	0.092	0.762
B3	30	321.9 / 15.9	229.9 / 7.1	116.7 / 72.5	0.793	0.159	0.048	0.061	0.799
B4	30	135.3 / 2.4	225.7 / 11.4	33.7 / 78.4	0.926	0.053	0.021	0.023	0.943
B5	30	323.4 / 13.2	231.7 / 7.1	114.3 / 74.9	0.865	0.116	0.018	0.021	0.866
B6	30	313.3 / 0.1	43.3 / 5.5	222.5 / 84.5	0.694	0.283	0.023	0.033	0.592
C1	30	297.4 / 8.3	35.5 / 44.1	199.1 / 44.7	0.835	0.104	0.061	0.073	0.875
C2	30	296.7 / 4.1	27.5 / 10.9	186.5 / 78.3	0.693	0.212	0.096	0.139	0.694
C3	30	293.2 / 6.2	200.7 / 22.0	38.1 / 67.0	0.474	0.312	0.214	0.451	0.342
C4	30	292.0 / 8.6	22.4 / 2.3	127.4 / 81.1	0.634	0.276	0.09	0.142	0.565
C5	30	295.5 / 9.0	143.1 / 79.9	26.3 / 4.6	0.736	0.153	0.112	0.152	0.792
C6	30	257.9 / 10.0	166.0 / 10.3	31.3 / 75.6	0.536	0.357	0.107	0.200	0.334
D1	30	138.7 / 6.9	230.3 / 12.7	20.9 / 75.6	0.622	0.316	0.063	0.101	0.492
D2	30	114.9 / 2.7	206.1 / 23.8	18.7 / 66.0	0.761	0.179	0.06	0.079	0.765
D3	30	265.6 / 14.0	358.5 / 11.5	126.5 / 71.8	0.543	0.305	0.151	0.278	0.438
D4	30	125.0 / 7.4	224.0 / 50.3	29.1 / 38.7	0.607	0.253	0.14	0.231	0.583
D5	30	288.7 / 7.1	193.0 / 38.7	27.4 / 50.5	0.718	0.183	0.099	0.138	0.745
D6	30	297.2 / 7.5	29.1 / 14.5	180.7 / 73.6	0.789	0.12	0.091	0.115	0.848
E1	30	301.5 / 3.1	33.1 / 28.0	205.7 / 61.8	0.828	0.103	0.069	0.083	0.876
E2	30	292.7 / 5.1	23.7 / 11.8	179.7 / 77.1	0.84	0.1	0.06	0.071	0.881
E3	30	302.6 / 8.0	42.9 / 51.9	206.6 / 37.0	0.886	0.066	0.047	0.053	0.926
E4	30	300.2 / 7.7	208.3 / 13.2	59.7 / 74.7	0.936	0.05	0.013	0.014	0.947
E5	30	297.3 / 5.0	27.3 / 0.1	118.1 / 85.0	0.86	0.115	0.024	0.028	0.866
T1	40	315.8 / 32.8	217.9 / 12.1	110.4 / 54.5	0.654	0.228	0.118	0.180	0.651
T2	40	302.4 / 10.2	33.4 / 5.6	151.9 / 78.3	0.684	0.227	0.09	0.132	0.668
T3	40	297.9 / 9.2	28.6 / 4.8	145.8 / 79.6	0.698	0.259	0.043	0.062	0.629
T4	40	323.5 / 25.9	221.9 / 22.4	96.6 / 54.6	0.501	0.319	0.18	0.359	0.363
T5	40	272.7 / 12.4	4.0 / 6.0	119.4 / 76.2	0.599	0.332	0.07	0.117	0.446
T6	40	297.0 / 9.7	28.2 / 7.3	154.5 / 77.8	0.754	0.225	0.02	0.027	0.702
X1	30	290.7 / 1.5	21.3 / 21.4	196.8 / 68.6	0.858	0.091	0.051	0.059	0.894
X2	30	280.2 / 4.8	12.7 / 27.0	180.9 / 62.5	0.775	0.153	0.072	0.093	0.803
X3	30	327.2 / 0.7	237.0 / 12.6	60.5 / 77.4	0.482	0.413	0.105	0.218	0.143
X4	30	279.6 / 9.3	186.9 / 15.8	38.9 / 71.5	0.635	0.288	0.077	0.121	0.546
X5	30	297.7 / 2.0	27.9 / 4.0	180.9 / 85.5	0.761	0.195	0.044	0.058	0.744
X6	30	295.8 / 2.1	205.2 / 18.2	32.1 / 71.6	0.709	0.182	0.109	0.154	0.743
X7	30	304.2 / 1.7	214.1 / 4.9	53.0 / 84.8	0.75	0.136	0.114	0.152	0.819
Y1	30	125.0 / 0.3	215.2 / 33.8	34.6 / 56.2	0.775	0.192	0.032	0.041	0.752
Y2	30	311.3 / 0.7	41.9 / 38.5	220.4 / 51.5	0.739	0.198	0.063	0.085	0.732
Y3	30	296.0 / 2.8	205.5 / 10.3	40.9 / 79.4	0.89	0.093	0.017	0.019	0.896
Y4	30	311.8 / 4.8	221.4 / 5.2	84.2 / 82.9	0.762	0.153	0.084	0.110	0.799
Y5	30	305.4 / 1.9	35.8 / 13.8	207.8 / 76.1	0.786	0.151	0.063	0.080	0.808
Y6	30	295.9 / 4.9	26.1 / 1.8	136.0 / 84.7	0.88	0.104	0.016	0.018	0.882
Z1	30	310.8 / 0.4	220.5 / 32.9	41.4 / 57.0	0.85	0.096	0.054	0.064	0.887
Z2	30	317.6 / 4.6	49.1 / 18.0	213.9 / 71.4	0.741	0.161	0.098	0.132	0.783
Z3	30	134.2 / 0.8	224.3 / 7.0	37.3 / 83.0	0.862	0.098	0.039	0.045	0.886
Z4	30	311.8 / 9.9	215.1 / 33.9	55.8 / 54.3	0.9	0.063	0.037	0.041	0.930
Z5	30	140.2 / 5.8	41.1 / 57.2	233.9 / 32.1	0.728	0.155	0.116	0.159	0.787

Supplementary Table 1. A summary of clast macrofabric data for all analysed samples in the study area.

	All	>2 mm	1-2 mm	0.5-1mm	250-500 µm	< 250 µm
**V_1**	01/306	01/299	05/312	02/301	01/305	02/301
**S_1**	0.46	0.53	0.50	0.47	0.46	0.47
**S_2**	0.41	0.38	0.38	0.42	0.41	0.42
**S_3**	0.13	0.09	0.12	0.11	0.13	0.11
n	8383	51	349	1973	4655	1355

Supplementary Table 2. Sample C1T CT fabric eigenvector and eigenvalues.
